# Aggression, Moral Disengagement and Empathy. A Longitudinal Study Within the Interpersonal Dynamics of Bullying

**DOI:** 10.3389/fpsyg.2021.703468

**Published:** 2021-09-10

**Authors:** Daniel Falla, Eva M. Romera, Rosario Ortega-Ruiz

**Affiliations:** Department of Psychology, Universidad de Cordoba, Cordoba, Spain

**Keywords:** aggression, moral disengagement, empathy, bullying, longitudinal design

## Abstract

Moral disengagement and empathy have been linked to aggression in traditional bullying. A number of longitudinal studies have focused on how these variables predict aggressive behavior within the dynamics of bullying. However, no conclusive results have been produced as to whether aggressive behavior in bullying can predict lower levels of empathy, and to date, no studies have explored in depth the mediating role of moral disengagement strategies in this relationship, which is the aim of this study. A total of 1,810 students (51.0% girls; *M*_age_ = 14.50; *SD* = 1.05) completed a survey in three waves at 6-month intervals. The results showed that aggressive behavior in bullying at Time 1 was inversely related to affective and cognitive empathy at Time 3. Minimization of responsibility, distortion of consequences and dehumanizing mediated in the aggressive behavior exhibited by the bullying aggressors and in cognitive empathy, while cognitive restructuring and the distortion of consequences mediated in affective empathy. We discuss the impact on moral and emotional sensitivity of the continued aggression occurring in the interpersonal dynamics of bullying, as well as the relationship between certain strategies of moral disengagement and the different types of empathy. We also comment on the need to design intervention programs to address the lowering of moral criteria and empathy in young people and adolescents involved in traditional bullying.

## Introduction

Aggressive behavior is a pattern of conduct whose adaptive origin is based on neurophysiological conditions, which are in turn modulated by processes of socialization (Blair, 2010). As a result, in most cases, aggressive behavior is controlled through cognitive and socio-affective processes which are derived from the competences which our brain uses in a global and coordinated way in its normal functions (Preckel et al., 2018). In this context, neuroscience has highlighted the key role played by empathy in regulating the processes which control aggressive impulses. Empathy, defined as the competence to register, recognize and experience the feelings and emotions of others (Weisz and Cikara, 2020), is a human characteristic, which like all the basic components of social behavior, is modulated and in most cases optimized for cognitive, emotional and socio-moral development throughout childhood and adolescence (see the systematic review by Silke et al., 2018). It is unquestionable that empathy plays a major role in the lives of groups, fostering the establishment of warm, affectionate and civic social relationships which help to stabilize the ecosystem of coexistence in which the development and learning processes take place (Ortega-Ruiz, 2020).

Studies in morality and aggressive behavior (Molchanov, 2014; Romera et al., 2019a) also point to the role of empathy in sensitivity and the recognition of aggression in bullying as an immoral act (Ortega-Ruiz, 2010). A growing number of studies have pointed out the relationship of the mechanisms of moral disengagement in acts of bullying, and that these cognitive processes can be activated in situations and interpersonal behavior where a moral judgment is required (see the meta-analysis by Killer et al., 2019 and the study into low levels of empathy by Haddock and Jimerson, 2017). Cross-sectional studies suggest that the experiences of being an aggressor toward one's peers, within the complex dynamics of bullying, lead to a greater indifference toward the victims' feelings compared to the moral sensitivity reported by those who are not aggressors toward their peers (Romera et al., 2019b).

### Aggression in Bullying and Moral Disengagement

Bullying is considered an intentional phenomenon involving persistent, unjustified aggression, and it is clear that this repeated abuse both damages the victim and lessens the aggressor's sensitivity and moral criteria, to such an extent that they become aware that what they are doing is morally reprehensible (Ortega-Ruiz, 2020). Aggressors use physical or psychological superiority to intimidate, mistreat and ultimately physically attack their victims in different ways, ranging from insulting or hitting to more sophisticated, relational forms of bullying, such as social exclusion (Menesini and Salmivalli, 2017). The prevalence figures indicate that about 36% of boys and girls bully their schoolmates with some frequency (Modecki et al., 2014), with boys being the most commonly involved and the most frequent perpetrators, although this male preponderance decreases in early adolescence (Smith et al., 2019).

It is undeniable that the unjustified, intentional and repetitive aggression that occurs in the dynamics of bullying includes ethical elements: in order to justify its repetition, the aggressor's repeated behavior and the roles of aggressor, victim and spectator, it is necessary to take a cynical view and deny the evidence that this aggression harms the victim. In this context, the definition of moral disengagement mechanisms proposed by Bandura (2002) is a key construct to help us progress in our understanding of the morally complex dynamics of the phenomenon of bullying. Bandura distinguished eight disengagement mechanisms, which were grouped into four domains or strategies as follows: (a) cognitive restructuring, which allows offenders to interpret behavior that is clearly immoral as fair or reasonable; (b) minimization of responsibility, which consists of disregarding or transferring responsibility for one's own antisocial actions; (c) distortion of the consequence, which permits the person committing immoral acts not to fully consider the impact their actions have on others; and (d) dehumanization, which is used to reject, undervalue or even blame the victim for what is happening. This model of four strategies of moral disengagement is particularly apt for understanding the unethical dimension of the type of aggression which occurs in the dynamics of bullying. A large number of scientific works have used this model (see meta-analysis by Gini et al., 2014; Killer et al., 2019) and some studies have revealed the possible socio-cultural intricacies of these strategies. For instance, according to Pornari and Wood (2010) in the cultural context of the United Kingdom, one common strategy is to minimize responsibility and the mechanisms related to cognitive restructuring, and to utilize euphemistic language to justify the facts; Scandinavian studies have also observed attribution of blame to the victim in order to justify such conduct (Thornberg and Jungert, 2014; Bjärehed et al., 2020). In Poland, mechanisms related to cognitive restructuring (advantageous compassion, euphemistic labeling and moral justification) and distortion of consequences were associated with the perpetration of bullying (Zych and Llorent, 2019). In Australia, Runions et al. (2019) pointed out that bullies commonly made use of the mechanisms of minimizing responsibility, distorting consequences and euphemistic labeling. In Spain, Romera et al. (2020) found that the disengagement mechanisms associated with bullying were dehumanization, distortion of consequences and cognitive restructuring, and reported that this last strategy was the one most closely linked to the aggressor's behavior.

Previous studies, taking the mechanism of moral disengagement as a one-dimensional construct, have shown that higher scores in perpetration in bullying predict higher scores in moral disengagement (Obermann, 2013; Thornberg et al., 2019). However, to date, no longitudinal studies have explored the possible influence of aggressive behavior within bullying on the different mechanisms of moral disengagement.

### Aggression and Empathy in the Bullying Phenomenon

As stated above, empathy has been defined as the human competence to recognize and experience the feelings and emotions of others. Traditionally, two types of empathy have been identified, depending on the importance of the more cognitive-rational or affective-emotional aspects in the process of putting oneself in another person's position. These types have been associated with two differential kinds of neural processing: cognitive and affective (Healey and Grossman, 2018), which do not act completely independently, but which can be differentiated behaviourally. The type known as affective empathy, which involves elements of emotional contagion, in which one is “infected” by another's emotions (Cuff et al., 2016), appears to involve subcortical structures such as the limbic lobe (Derntl et al., 2010), while cognitive empathy seems to stimulate the activation of the pre-frontal and ventromedial cortex (Decety, 2011), which permits a certain prevalence of reflective and perhaps rational thinking. Thus, cognitive empathy allows us to understand not only the emotions and feelings of others, in line with what has been called the Theory of Mind (Healey and Grossman, 2018), but also to realize that the other person is a human being similar to oneself and who, therefore, can be expected to think, feel and behave as one does, or decide not to. In the face of conflicts of interest and rivalries, empathy allows us, on the one hand, to sympathize with the feelings of the sufferer and, on the other, to hypothesize to what extent the response of the other, in the heat of the conflict, will be aggressive or peaceful. Thus, it is to be expected that empathy which is both cognitive and affective, socialized and adjusted to socio-moral norms works as a control and modulation mechanism in conflict dynamics (Klimecki, 2019) which may include aggressive behavior (Tampke et al., 2020).

Research has revealed that interpersonal situational contexts significantly influence an individual's empathic processing (Cheng et al., 2017). In socially complex interpersonal dynamics such as bullying, where sustained hostility plays an important role, empathy and aggression seem to interact (van Noorden et al., 2015). In addition, studies based on the cycle of violence (Widom, 1989) have shown how basic social skills, fundamental to the development of empathy, are impaired in hostile contexts where there is exposure to ongoing violence and abuse (Heleniak and McLaughlin, 2020). Similarly, social cognitive theory points out how context can affect socio-cognitive reasoning and thus affective processing, including empathy (Bandura, 2002). Recent meta-analyses have shown that low empathy is related to a higher tendency toward aggression (Zych et al., 2019), although other meta-analyses (Vachon and Lynam, 2013) have produced conflicting results, albeit among adults. In longitudinal studies, some researchers have found that aggression in bullying influences cognitive empathy (Williford et al., 2016), while other studies found no direct relationship between bullying and empathy (Walters and Espelage, 2018) and others showed that, in bullying, empathy and aggression are bidirectionally related (Stavrinides et al., 2010). In short, although cross-sectional studies indicate that aggression and empathy are related, more longitudinal work remains to be done to test whether bullying may be related to lower affective and cognitive empathy scores in the medium to long term.

### Aggression in Bullying, Moral Disengagement and Empathy

According to the general aggression model (GAM) individuals behave aggressively due to the interaction of personal and situational factors, internal states and outcomes of evaluation and decision-making processes (Dewall et al., 2011). This multi-causal influence is also supported by social-cognitive theory that describes how there is also a bidirectional and reciprocal relationship between morality and aggression (Bandura, 2002). That is, hostile contexts can affect the moral judgement of individuals. Thus, aggression in bullying, as mentioned above, predicts higher scores on moral disengagement (Obermann, 2013; Thornberg et al., 2019). Considering the relationship described by Bandura (2002) between morality and affective processes such as empathy, the studies coming from neuroscience describing the existence of a social brain where morality and empathy are interconnected (Detert et al., 2008; Chen et al., 2018) and the evidence from developmental and educational psychology that supports an inverse relationship between moral disengagement and affective and cognitive empathy (Haddock and Jimerson, 2017), it would be plausible to hypothesize that aggression may also affect these socioemotional and socio-cognitive skills in the medium term. Thus, mechanisms of moral disengagement, in addition to preventing individuals from feeling negative emotions when committing transgressions (Mazzone et al., 2019), may lead to a decrease in affective and cognitive empathy. For example, in a previous study it was found that young people who had experiences as aggressors, tended to point to victims as indifferent to aggression (Romera et al., 2019b). This tendency may suggest that when as children engage in bullying, their ability or interest in taking the perspective of others (cognitive empathy) may be diminished (Haddock and Jimerson, 2017). There is also work indicating that a lack of emotional contagion or disconnection may occur among schoolchildren, as a type of adaptive response to avoid feeling negative emotions in maladaptive situations (Herrera-López et al., 2017).

Although there seem to be no studies examining which mechanisms of moral disengagement are most affected by aggression in bullying, some work has found a particularly important role played by cognitive restructuring in aggression in bullying (Falla et al., 2020). It therefore seems sensible to hypothesize that justifying or normalizing aggressive behavior, as well as inhibiting the negative emotions that transgressing the social norm would entail (Mazzone et al., 2019), could prevent recognition of the victim's emotions and also emotional contagion from occurring, even to the point of experiencing positive emotions for aggressing (Perren et al., 2012). Similarly, for some authors, ignoring or distorting consequences allows aggressors to disassociate themselves from the emotional harm of harmful actions, which may affect both empathies, so that the aggressor may infer that the victim accepts the aggressive behavior as a joke (Runions and Bak, 2015). Finally, attributing blame to the victim or dehumanizing the victim, in addition to holding the victim responsible for the behavior, leads to invalidating the victim's emotions and prevents emotional contagion. In this way, perpetrators become less likely to empathize with the victim and instead more motivated to hurt them (Haslam and Loughnan, 2014).

### Aims of the Study

There is evidence of a relationship between aggressive behavior in bullying and the mechanisms of moral disengagement (Killer et al., 2019), as well as between these cognitive processes and empathy (Haddock and Jimerson, 2017). However, as yet, there is no proof whether moral disengagement strategies exert any mediating effect between aggression in bullying and cognitive and affective empathy, and whether this impact is sustained over time. The aims of this study are: (1) to explore whether there is a relationship between aggression in bullying in Time 1 and cognitive and affective empathy in Time 3; (2) to examine whether certain strategies of moral disengagement exert any mediating impact between aggressive behavior and the levels of cognitive and affective empathy over all three time measures. For this, we followed Mediation Model 4 (Hayes, 2013) and the following hypotheses were proposed:

H1. There will be a negative relationship between aggressive behavior in bullying and cognitive and affective empathy, which is sustained significantly over time.

H2. The strategies of moral disengagement, cognitive restructuring, distortion of consequences and dehumanization will mediate the relationship between aggressive behavior in bullying and cognitive and affective empathy.

## Methods

### Participants

Thirteen schools in southern Spain (five urban and eight rural) were selected for accessibility using non-probabilistic sampling (Singleton and Straits, 2004). Although the data came from different classrooms and centers, they were taken as non-nested due to the statistical analyses used. The longitudinal study included three time waves, each six months apart, with a total period of one year between the first and the third. Time 1, between April and May 2018, involved 2,360 students (50.1% girls; *M*_age_ = 13.58; *SD* = 1.13) with the following distribution by school years: 7th (35.4%), 8th (33.6%) and 9th (31.0%). Time 2 took place between October and November 2018, with a retention rate of 86.06% (*N* = 2031) with (51.2% girls; *M*_age_ = 13.97; *SD* = 1.04), while the distribution by school years was: 7th (2.8%), 8th (37.2%), 9th (31.0%) and 10th (29.0%). In Time 3, the questionnaires were completed between April and May 2019, with a retention rate of 76.69% (*N* = 1810) with (51.0% girls; *M*_age_ = 14.50; *SD* = 1.05) and with the following distribution by school years: 7th (1.9%), 8th (37.8%), 9th (30.6%) and 10th (29.7%). The decrease in the total sample between waves was due to the fact that some schoolchildren did not attend on the day the survey was administered and others had changed school. Logistic regression was performed to check whether the analytical longitudinal sample was representative of the total sample, and no significant differences were found (all *ps* > 0.05) in the study variables between any of the three time periods.

### Instruments

Empathy was measured using *The Basic Empathy Scale* (Jolliffe and Farrington, 2006). This scale contains 20 items, with a Likert scale from one to five (1 = *strongly disagree* to 5 = *strongly agree*) distributed in two factors: cognitive empathy (nine items) (e.g., “I can understand my friend's happiness when he/she does something well”) and affective empathy (eleven items) (e.g., “After spending time with a friend who is upset about something, I usually feel sad”). The reliability analyses were acceptable for both cognitive empathy (ω_*T*1_ = 0.75, ω_*T*2_ = 0.77, ω_*T*3_ = 0.80) and affective empathy (ω_*T*1_ = 0.77, ω_*T*2_ = 0.77, ω_*T*3_ = 0.78).

Aggression in bullying was measured using the Spanish version of *the European Bullying Intervention Project Questionnaire* (EBIPQ) (Ortega-Ruiz et al., 2016). This scale is made up of 14 items, referring to the last two months and divided into two factors, and is scored on a Likert scale from 0 to 4 (0 = *no*; 1 = *yes, once or twice*; 2 = *yes, once or twice a month*; 3 = *yes, about once a week*; 4 = *yes, more than once a week*). For the current study, only the “aggression” factor was used, which is made up of seven items (e.g., “I have excluded or ignored someone”). Omega coefficients were good for all three time periods (ω_*T*1_ = 0.81, ω_*T*2_ = 0.81, ω_*T*3_ = 0.78).

The mechanisms of moral disengagement were measured using the *Mechanisms of Moral Disengagement Scale* (Caprara et al., 1996). The version used consisted of 24 items with five Likert-type response options, from 1 to 5 (1 = *strongly disagree*; 2 = *partially agree*; 3 = *generally agree*; 4 = *strongly agree*; 5 = *totally agree*), which were divided into four factors. The factorial structure of this instrument has been confirmed by Pozzoli et al. (2012). The domains were: cognitive restructuring (e.g., “It's okay to use force against a partner who insults your family”), minimizing responsibility (e.g., “You can't blame kids for swearing at their peers because most of their friends do it”), distorting the consequences (e.g., “Making fun of a classmate is not really hurting him”) and dehumanizing (e.g., “There's nothing wrong with treating someone badly if they behave in a contemptible way”). The reliability analyses were acceptable: cognitive restructuring (ω_*T*1_ = 0.83, ω_*T*2_ = 0.83, ω_*T*3_ = 0.85); minimization of responsibility (ω_*T*__1_ = 0.70, ω_*T*2_ = 0.73, ω_*T*3_ = 0.75); distortion of consequences (ω_*T*1_ = 0.59, ω_*T*2_ = 0.60, ω_*T*3_ = 0.66); and dehumanization (ω_*T*1_ = 0.76, ω_*T*2_ = 0.76, ω_*T*3_ = 0.79).

### Procedure

The Ethics Committee (who remained anonymous) previously approved the project used to carry out this study. First, we contacted the secondary schools to explain the objectives of the study and request their participation. This was agreed by the schools' councils and, next, letters of consent were sent out to the families. Once permission had been obtained from the schools and families, the dates for conducting the survey were set.

The survey was administered in the classroom: one of the researchers explained the procedure and reminded the children of the anonymous, voluntary nature of the study. In addition, the researcher explained how to fill in the code required to be able to carry out the longitudinal study. Any children who did not want to fill in the questionnaires remained in the classroom, and the children were given approximately 30 min to complete the questionnaires.

### Data Analysis

The descriptive analyses included means, standard deviations, bivariate correlations and Student's *t* and Cohen's *d tests* to determine gender differences and effect size, using IBM SPSS Statistics Version 26 (IBM, Armonk, NY, USA). The mediation analysis was performed using the PROCESS v3.4 macro for SPSS (SPSS Inc., Chicago, IL, USA), and all the variables used were standardized to be able to make comparisons of the effects. Model 4 was used following Hayes (2013), and the MacKinnon (2008) four-step procedure was followed. The variables used were aggression in bullying at Time 1 as a predictor variable, cognitive restructuring at Time 2 as the first mediator, minimization of responsibility as the second mediator, distortion of consequences as the third mediator, dehumanization as the fourth mediator, cognitive empathy as a dependent variable for the first model and affective empathy as a dependent variable for the second model. Gender and age were used as covariates in all the analyses.

Indirect effects were tested with the bootstrapping method, in which the values were considered significant when the confidence intervals did not include zero. This method is optimal for linear hypotheses when the variables do not have a normal distribution (Chernick, 2008). The relationship between the independent and dependent variable enabled us to find the total effect, while the mediation effect was calculated between the indirect effect and the total effect (Wen and Fan, 2015).

## Results

### Descriptive Results

The correlations between all the study variables were checked for the three time periods. A direct relationship was found between all three, except between age and the bullying-perpetration variables T2 and T3, and between minimization of responsibility T2 and T3 and gender (see Supplementary Material). The assumption of multicollinearity was not violated since the VIF was <2.42 in all variables. Similarly, the Student's *t tests* allowed us to verify the existence of significant differences between boys and girls for all the study variables. The girls scored higher in the two dimensions of empathy while boys obtained higher marks for the rest of the variables. The effect sizes were low to moderate (see Table 1).

**Table 1:** Means, standard deviations and differences by gender for all variables.

	**Sample**		**Boys**		**Girls**		
	***M***	***SD***	***M***	***SD***	***M***	***SD***	***t***	***d***
BP T1	0.27	0.42	0.32	0.49	0.21	0.35	5.23^**^	0.26
CR T1	1.53	0.58	1.69	0.64	1.39	0.48	10.89^**^	0.53
MR T1	1.79	0.72	1.86	0.77	1.72	0.66	4.18^**^	0.20
DC T1	1.38	0.57	1.50	0.67	1.28	0.42	7.89^**^	0.40
DH T1	1.49	0.61	1.61	0.70	1.37	0.48	8.36^**^	0.42
CE T1	4.07	0.55	3.94	0.56	4.19	0.51	−9.69^**^	0.47
AE T1	3.55	0.65	3.32	0.66	3.76	0.57	−14.02^**^	0.72
BP T2	0.19	0.37	0.25	0.45	0.14	0.28	5.98^**^	0.30
CR T2	1.51	0.57	1.67	0.65	1.37	0.45	11.27^**^	0.54
MR T2	1.72	0.71	1.77	0.75	1.68	0.66	2.68^*^	0.13
DC T2	1.36	0.56	1.45	0.64	1.28	0.46	6.54^**^	0.31
DH T2	1.46	0.59	1.58	0.68	1.36	0.47	7.57^**^	0.38
CE T2	4.08	0.56	3.94	0.60	4.21	0.50	−9.92^**^	0.49
AE T2	3.55	0.64	3.30	0.63	3.76	0.57	−15.26^**^	0.77
BP T3	0.20	0.36	0.25	0.42	0.16	0.29	5.03^**^	0.25
CR T3	1.53	0.60	1.69	0.67	1.39	0.48	10.52^**^	0.52
MR T3	1.71	0.71	1.76	0.76	1.67	0.65	2.76^*^	0.13
DC T3	1.35	0.56	1.46	0.65	1.25	0.43	8.07^**^	0.38
DH T3	1.45	0.60	1.58	0.69	1.34	0.47	8.13^**^	0.41
CE T3	4.09	0.56	3.96	0.57	4.21	0.51	−9.50^**^	0.46
AE T3	3.56	0.64	3.32	0.63	3.78	0.56	−15.57^**^	0.77

*M, mean; SD, standard deviation; t, student's t; d, cohen's d; AG, bullying perpetration; CR, cognitive restructuring; MR, minimizing responsibility; DC, distorting consequences; DH, dehumanizing; CE, cognitive empathy, AE, affective empathy;*

* *p < 0.05;*

** *p < 0.001*.

### Mediation Analysis for Cognitive Empathy Model

**Figure 1:**
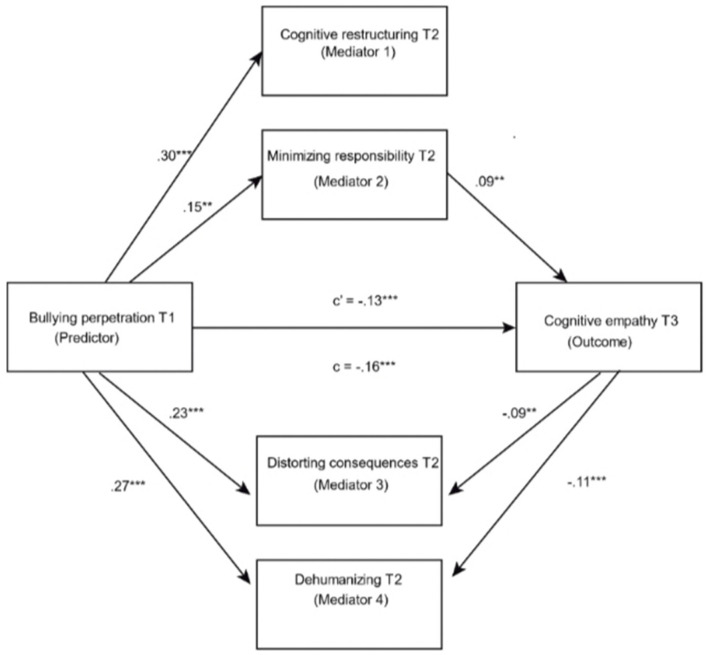
Results in the pathways of the model. **p < 0.01; ***p < 0.001.

The mediation analyses were carried out using Model 4 (Hayes, 2013), and proved that the effect of bullying-perpetration in T1 (predictor variable) on cognitive empathy in T3 (dependent variable) was mediated by cognitive restructuring (mediator 1), minimization of responsibility (mediator 2), distortion of consequences (mediator 3) and dehumanization (mediator 4) was significant: *F*_(7, 1469)_ = 22.78; *R*^2^ = 0.10; *p* < 0.001. The data indicated a direct and negative relationship of bullying-perpetration in T1 on cognitive empathy in T3 (β = −0.16, *t* = −6.09, *p* < 0.01). In step 2, there was a direct, positive association of bullying perpetration with cognitive restructuring (β = 0.30, *t* = 12.91, *p* < 0.01), minimization of responsibility (β = 0.15, *t* = 5.61, *p* < 0. 01), distortion of the consequences (β = 0.23, *t* = 8.92, *p* < 0.01) and dehumanization (β = 0.27, *t* = 10.35, *p* < 0.01). In step 3, a direct and positive relationship was found for minimization of responsibility in T2 with cognitive empathy in T3 (β = 0.09, *t* = 2.66, *p* < 0.01), together with a direct, negative relationship of the distortion of the consequences (β = −0.09, *t* = −2.60, *p* < 0.01) and dehumanization (β = −0.11, *t* = −2.74, *p* < 0.01) on cognitive empathy T3. In addition, a negative relationship was found between bullying perpetration and cognitive empathy (β = −0.13, *t* = −4.44, *p* < 0.01) (see Figure 1).

The percentile bootstrap method with bias correction indicated a positive relationship of the indirect effect of bullying perpetration T1 on cognitive empathy in T3 through the minimization of responsibility (β = 0.013, 95% CI = [0.003, 0.03]) and a negative effect by the pathways of distortion of consequences (β = −0.02, 95% CI = [−0.04, −0.004]) and dehumanization (β = −0.03, 95% CI = [−0.05, −0.004]). Thus, the mediation effect was 7.93% in minimization of responsibility, 12.93% in distortion of the consequences and 17.32% in dehumanization.

### Analysis of Mediation Effect for the Affective Empathy Model

**Figure 2:**
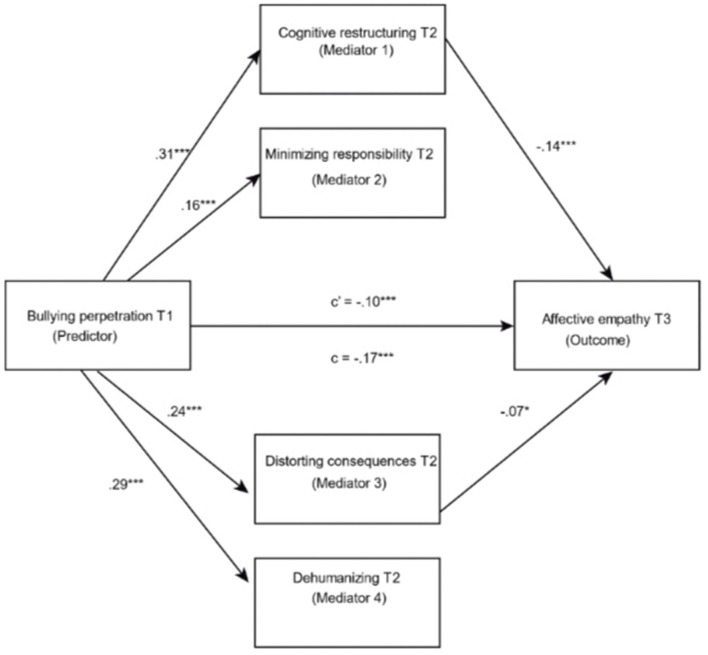
Results in the pathways of the model. *p < 0.05; **p < 0.01; ***p < 0.001.

Model 4 (Hayes, 2013) was also significant: *F*_(7, 1457)_ = 49.12; *R*^2^ = 0.19; *p* < 0.001 for the T1 perpetration bullying model on affective empathy in T3 mediated by cognitive restructuring, minimization of responsibility, distortion of consequences and dehumanization. Firstly, the bullying perpetration variable in T1 exerted a direct, negative relationship on cognitive empathy in T3 (β = −0.17, *t* = −6.22, *p* < 0.01). Secondly, the predictor variable had a direct, positive relationship with the four mediators: cognitive restructuring (β = 0.31, *t* = 12.62, *p* < 0.01), minimization of responsibility (β = 0.16, *t* = 5.58, *p* < 0.01), distortion of consequences (β = 0.24, *t* = 8.99, *p* < 0.01) and dehumanization (β = 0.29, *t* = 10.71, *p* < 0.01). Thirdly, the data showed a direct relationship for cognitive restructuring (β = −0.14, *t* = −3.45, *p* < 0.01) and the distortion of consequences (β = −0.07, *t* = −2.14, *p* < 0.01) with affective empathy, while bullying perpetration also correlated negatively with affective empathy (β = −0.10, *t* = −3.57, *p* < 0.01) (see Figure 2).

The analyses using the percentile bootstrap method with correction for bias showed a negative relationship for the indirect effect of bullying perpetration T1 on affective empathy in T3 via the cognitive restructuring pathways (β = −0.04, 95% CI = [−0.07, −0.02]) and distortion of consequences (β = −0.02, 95% CI = [−0.04, −0.001]). In this way, cognitive restructuring accounted for 25.21% of the mediation effect, while distortion of consequences contributed 10.47%.

## Discussion

The prolonged aggression that occurs among young people and adolescents, in which the bullying aggressor dominates in a cruel, unjustified way, producing a number of negative effects on the victim's moral integrity, constitutes immoral behavior, as it contradicts the general tendency toward socialization, which aims to find ways of avoiding violence following the ethical principles of society. In turn, the process of socialization appears to rely on human competence in order to be sensitive to the feelings of others, through a cognitive and affective process known as empathy. Empathy has especially attracted the interest of researchers into bullying as a protective factor against aggressive behavior (Garandeau et al., 2021). In addition, neuroscientific studies using neuroimaging inform us that moral judgments and empathic processing depend on past experiences (see meta-analysis by Bzdok et al., 2012). Similarly, it has been found that empathy is susceptible to the situational social context (Cheng et al., 2017) and that empathy, moral development and aggressive behavior are closely related (Blair, 2010). Based on these neurophysiological and psychological theoretical foundations, therefore, we hypothesized in this work that in the hostile, sustained context of bullying, the perpetrators will increase their mechanisms of moral disengagement and this will be related to lower levels of affective and cognitive empathy.

The first of the hypotheses was confirmed. It was shown that the aggressive behavior that occurs in bullying at Time 1 is related to low cognitive and affective empathy at Time 3. We know from research in the field of neurosciences that humans are sometimes able to avoid the emotional contagion which occurs through affective empathy to protect themselves from negative emotions such as guilt, pain or anguish (Lamm et al., 2007; Bensalah et al., 2016). This could happen in the case of the perpetrators in bullying, who end up dissociating themselves from the emotional contagion derived from affective empathy. However, these aggressors may also end up having less understanding of other people's thoughts and feelings, as Williford et al. (2016) found in their longitudinal study, and as found by cross-sectional studies in elementary school students who had participated in bullying as aggressors indicated (when represented in vignettes showing aggressor-victim dynamics), these experiences were not related to the suffering they were made to feel (Romera et al., 2019b). In other words, these findings suggest that the continuous aggression over time found in this type of bullying can lead to the aggressors to lower both types of empathy toward the victim, which clouds their judgment about the suffering that they are causing the other person. This lack of emotional sensitivity, which has been studied as emotional disengagement in other works on empathy and normative adjustment of young people and adolescents (Herrera-López et al., 2017), as well as in research on cognitive neuroscience (Decety and Svetlova, 2012), could be a key factor to explain both the sustained repeated aggression toward the victim and the activation of a mechanism to disengage from the critical moral judgment about the behavior itself: that is, a mechanism of moral disengagement.

The second hypothesis was partly confirmed. The distortion of consequences, whose relationship with aggressive behavior in bullying has already been proved in cross-sectional studies (Runions et al., 2019; Romera et al., 2020), was the only mechanism of moral disengagement which mediated between the sustained aggression and the levels of cognitive and affective empathy, throughout the three periods of this longitudinal study. It is known that moral disengagement strategies are used to evade uncomfortable feelings of guilt and moral responsibility in aggressive behavior. It may be that, in an attempt to avoid these feelings, they elude the emotional contagion and solidarity implicit in affective and cognitive empathy: to do this, the consequences of harm have to be distorted. The strategies of minimizing responsibility and dehumanizing the victim also mediated in the cognitive empathy scores. In other words, the act of avoiding responsibility for the aggression or attributing the blame to the characteristics of the victim end up influencing the interpretation made by the aggressors about the feelings and thoughts of the victims themselves. However, in the case of responsibility minimization, it is a mechanism that requires recognition of the harm done to another person and the attribution of responsibility to others in order to alleviate feelings of guilt, hence its possible positive relationship with mechanisms that involve knowing how another person feels. However, the relationship is weak and should be further explored in future research. Whereas, in the case of dehumanization, the relationship is negative, which could explain certain phenomena such as discriminatory bullying (Rodríguez-Hidalgo et al., 2019), in which the aggressors harass the victims for reasons of gender, disability, race or cultural ethnicity, and this attribution of dehumanization and guilt about their defects could reinforce the moral impunity of the aggressors. On the other hand, cognitive restructuring and the distortion of consequences mediated in the scores for affective empathy. It seems logical to assume that these two strategies of moral disengagement, which involve a cold, external attribution, allow the bully to avoid feeling sympathy and empathy with those who suffer at their hands and avoid the emotional contagion and solidarity that could result from it; however, at the same time, it uses moral cynicism to reinforce the behavior of repeated, sustained aggression over time. These findings are consistent with previous studies which point to the importance of moral emotions in motivating pro-social behavior and an individual's moral self-concept (Christner et al., 2020).

### Limitations and Practical Implications

This work has certain limitations which must be taken into account. The study sample was taken from a single country, so studies including samples from different countries could, firstly, confirm whether these processes are universal and, secondly, provide valuable information about the cultural element in processes which have an implicit moral criterion and shared values present in all interpersonal dynamics and, particularly, in bullying (Ortega-Ruiz, 2020). On the other hand, the use of self-reports as the sole source of data may lead to response bias, which could be resolved by conducting experimental or qualitative studies. Similarly, as this was an exploratory study and its purpose was to find out the medium-term interactions between aggressiveness in bullying, mechanisms of moral disengagement and affective and cognitive empathy, as well as the indirect effects of the mediating variables, a mediation analysis with the PROCESS macro was used. However, running path analyses using other software to deal with the non-normality of the variables and including both criterion variables in the same model, or even nesting the sample, could provide more robust results on these interactions. Also, the criterion variables, affective and cognitive empathy, were not controlled for at time 1. This is an important limitation that would have diminished the strength of the association between the variables, so we recommend that these limitations be addressed in future studies.

Despite the limitations, this study is a first step to consider the longitudinal interplay between aggression, moral disengagement strategies and empathy and provides relevant findings which further our knowledge in the complex interpersonal dynamics that take place in bullying, which, as stated above, is a clearly immoral, unfair and repetitive type of aggression. Although empathy has already been studied as a relevant protective factor in bullying (Garandeau et al., 2021), this work broadens this knowledge and argues that the low affective and cognitive empathy of the perpetrators of bullying possibly results from the continued experience of engaging in aggressive behavior which is clearly unfair and which infringes the general principles of socialization, which stress the importance of fair, respectful treatment toward others. If, as the data seem to show, the aggressors in bullying make use of mechanisms of moral disengagement without interruption over an extended period of time (remember here that bullying is not one specific event, but a persistent, repeated action), this will clearly lead to a lack of empathy. In the particular case of the mechanism for distorting the consequences for the victim, it is evident that an important cynical bias is at work in the moral criterion which mediates the main aspects of critical judgment. The findings of this work therefore show that the immorality and the deterioration of empathy experienced by the aggressors should also be addressed in specific programs as a consequence of the continuous aggression.

This work, in line with the contributions of neuroscientific research, highlights the close relationship between empathic processes and moral judgments, which can be especially useful in preventive and palliative intervention programs. For instance, mechanisms of moral disengagement focused on normalizing behavior and reducing consequences are linked to low affective empathy, so intervention programs could specifically focus on working on these aspects together in aggressors who show low affective empathy. On the other hand, cognitive empathy shows that specific work needs to be done to recognize the humanization of the victims and to encourage the self-recognition of responsibility for the harm caused to others.

## Conclusions

The results of this work reinforce the close ties between cognitive and affective empathy and moral disengagement strategies, and show that the deterioration of empathy and high levels of moral disengagement strategies may be the consequence of the repeated use of these strategies and the deterioration of empathic sensitivity. The combined action of both processes reinforcing each other could account for the fact that the profile of the aggressor in bullying is dangerously far from the expectations of socialization and therefore of the control over their own behavior which moral principles dictate. In a nutshell, encouraging young people and adolescents to develop more critical, ethical thinking which is more supportive toward others requires a major effort of emotional and moral sensitivity to generate motivation to repair the damage caused, which seems an unlikely outcome if the strategies of moral disengagement are stubbornly perpetuated, or are increased. Dialogue, the peaceful resolution of conflicts and the beneficial effect of good friendships within the framework of interpersonal relationships are some of the elements of the social context that can have a palliative effect. However, as shown in this work, continuous, uninterrupted aggression which is not controlled by the context seems to reinforce in the aggressor biased moral judgments which are disengaged from the sensitivity and empathy toward others that civilized socialization requires, and intervention programs should focus their preventive and palliative work on this area.

## Data Availability Statement

The raw data supporting the conclusions of this article will be made available by the authors, without undue reservation.

## Ethics Statement

The studies involving human participants were reviewed and approved by The Ethics Committee of Bioethics and Biosafety at University of Córdoba. Written informed consent to participate in this study was provided by the participants' legal guardian/next of kin.

## Author Contributions

All authors contributed to the interpretation of data, helped to draft, revise the manuscript, read, and approved the final manuscript.

## Funding

ER and RO-R were supported by project PSI2016-74871-R and DF, ER, and RO-R by project PSI2020-113911-RB-I00 (National Research Agency, Ministerio de Ciencia e Innovación, Spain).

## Conflict of Interest

The authors declare that the research was conducted in the absence of any commercial or financial relationships that could be construed as a potential conflict of interest.

## Publisher's Note

All claims expressed in this article are solely those of the authors and do not necessarily represent those of their affiliated organizations, or those of the publisher, the editors and the reviewers. Any product that may be evaluated in this article, or claim that may be made by its manufacturer, is not guaranteed or endorsed by the publisher.

## References

[B1] Bandura A. (2002). Selective moral disengagement in the exercise of moral agency. J. Moral Educ. 31, 101–119. 10.1080/0305724022014322

[B2] Bensalah L. Stefaniak N. Carre A. Besche-Richard C. (2016). The basic empathy scale adapted to French middle childhood: structure and development of empathy. Behav. Res. Methods 48, 1410–1420. 10.3758/s13428-015-0650-8 26424437

[B3] Bjärehed M. Thornberg R. Wänström L. Gini G. (2020). Mechanisms of moral disengagement and their associations with indirect bullying, direct bullying, and pro-aggressive bystander behavior. J. Early Adolesc. 40, 28–55. 10.1177/0272431618824745

[B4] Blair R. J. R. (2010). Empathy, moral development, and aggression: a cognitive neuroscience perspective, in Emotions, Aggression, and Morality in Children: Bridging Development and Psychopathology (Worcester, MA: American Psychological Association), 97–114. 10.1037/12129-005

[B5] Bzdok D. Schilbach L. Vogeley K. Schneider K. Laird A. R. Langner R.. (2012). Parsing the neural correlates of moral cognition: ALE meta-analysis on morality, theory of mind, and empathy. Brain Struct. Funct. 217, 783–796. 10.1007/s00429-012-0380-y 22270812 PMC3445793

[B6] Caprara G. V. Barbaranelli C. Vicino S. Bandura A. (1996). La misura del disimpegno morale in età evolutiva. Età Evolutiva 51, 18–29.

[B7] Chen C. Martínez R. M. Cheng Y. (2018). The developmental origins of the social brain: empathy, morality, and justice. Front. Psychol. 9:2584. 10.3389/fpsyg.2018.02584 30618998 PMC6302010

[B8] Cheng Y. Chen C. Decety J. (2017). How situational context impacts empathic responses and brain activation patterns. Front. Behav. Neurosci. 11:165. 10.3389/fnbeh.2017.00165 28928643 PMC5591329

[B9] Chernick M. R. (2008). Bootstrap Methods: A Guide for Practitioners and Researchers, 2nd Edn. Hoboken, NJ: Wiley.

[B10] Christner N. Pletti C. Paulus M. (2020). Emotion understanding and the moral self-concept as motivators of prosocial behavior in middle childhood. Cogn. Dev. 55:100893. 10.1016/j.cogdev.2020.100893

[B11] Cuff B. M. P. Brown S. J. Taylor L. Howat D. J. (2016). Empathy: a review of the concept. Emot. Rev. 8, 144–153. 10.1177/1754073914558466

[B12] Decety J. (2011). Dissecting the neural mechanisms mediating empathy. Emot. Rev. 3, 92–108. 10.1177/1754073910374662

[B13] Decety J. Svetlova M. (2012). Putting together phylogenetic and ontogenetic perspectives on empathy. Dev. Cogn. Neurosci. 2, 1–24. 10.1016/j.dcn.2011.05.003 22682726 PMC6987713

[B14] Derntl B. Finkelmeyer A. Eickhoff S. Kellermann T. Falkenberg D. I. Schneider F.. (2010). Multidimensional assessment of empathic abilities: neural correlates and gender differences. Psychoneuroendocrinology 35, 67–82. 10.1016/j.psyneuen.2009.10.006 19914001

[B15] Detert J. R. Treviño L. K. Sweitzer V. L. (2008). Moral disengagement in ethical decision making: a study of antecedents and outcomes. J. Appl. Psychol. 93, 374–391. 10.1037/0021-9010.93.2.374 18361639

[B16] Dewall C. N. Anderson C. A. Bushman B. J. (2011). The general aggression model: theoretical extensions to violence. Psychol. Violence 1, 245–258. 10.1037/a0023842

[B17] Falla D. Ortega-Ruiz R. Runions K. Romera E. M. (2020). Why do victims become perpetrators of peer bullying? Moral disengagement in the cycle of violence. Youth Society. 10.1177/0044118X20973702

[B18] Garandeau C. F. Laninga-Wijnen L. Salmivalli C. (2021). Effects of the KiVa anti-bullying program on affective and cognitive empathy in children and adolescents. J. Clin. Child Adolesc. Psychol. 1–15. 10.1080/15374416.2020.1846541 33448897

[B19] Gini G. Pozzoli T. Hymel S. (2014). Moral disengagement among children and youth: a meta-analytic review of links to aggressive behavior. Aggress. Behav. 40, 56–68. 10.1002/ab.21502 24037754

[B20] Haddock A. D. Jimerson S. R. (2017). An examination of differences in moral disengagement and empathy among bullying participant groups. J. Relat. Res. 8, 1–15. 10.1017/jrr.2017.15

[B21] Haslam N. Loughnan S. (2014). Dehumanization and infrahumanization. Annu. Rev. Psychol. 65, 399–423. 10.1146/annurev-psych-010213-115045 23808915

[B22] Hayes A. F. (2013). Introduction to Mediation, Moderation, and Conditional Process Analysis. New York, NY: Guilford.

[B23] Healey M. L. Grossman M. (2018). Cognitive and affective perspective-taking: evidence for shared and dissociable anatomical substrates. Front. Neurol. 9:491. 10.3389/fneur.2018.00491 29988515 PMC6026651

[B24] Heleniak C. McLaughlin K. A. (2020). Social-cognitive mechanisms in the cycle of violence: cognitive and affective theory of mind, and externalizing psychopathology in children and adolescents. Dev. Psychopathol. 32, 735–750. 10.1017/S0954579419000725 31407638 PMC7015789

[B25] Herrera-López M. Gómez-Ortiz O. Ortega-Ruiz R. Jolliffe D. Romera E. M. (2017). Suitability of a three-dimensional model to measure empathy and its relationship with social and normative adjustment in Spanish adolescents: a cross-sectional study. BMJ Open 7:e015347. 10.1136/bmjopen-2016-015347 28951400 PMC5623524

[B26] Jolliffe D. Farrington D. P. (2006). Development and validation of the basic empathy scale. J. Adolesc. 29, 589–611. 10.1016/j.adolescence.2005.08.010 16198409

[B27] Killer B. Bussey K. Hawes D. J. Hunt C. (2019). A meta-analysis of the relationship between moral disengagement and bullying roles in youth. Aggress. Behav. 45, 450–462. 10.1002/ab.21833 30900277

[B28] Klimecki O. M. (2019). The role of empathy and compassion in conflict resolution. Emot. Rev. 11, 310–325. 10.1177/1754073919838609

[B29] Lamm C. Batson C. D. Decety J. (2007). The neural substrate of human empathy: effects of perspective-taking and cognitive appraisal. J. Cogn. Neurosci. 19, 42–58. 10.1162/jocn.2007.19.1.42 17214562

[B30] MacKinnon D. P. (2008). Introduction to Statistical Mediation Analysis. New York, NY: Taylor & Francis Group.

[B31] Mazzone A. Yanagida T. Caravita S. C. S. Strohmeier D. (2019). Moral emotions and moral disengagement: Concurrent and longitudinal associations with aggressive behavior among early adolescents. J. Early Adolesc. 39, 839–863. 10.1177/2F0272431618791276

[B32] Menesini E. Salmivalli C. (2017). Bullying in schools: the state of knowledge and effective interventions. Psychol. Health Med. 22, 240–253. 10.1080/13548506.2017.1279740 28114811

[B33] Modecki K. L. Minchin J. Harbaugh A. G. Guerra N. G. Runions K. C. (2014). Bullying prevalence across contexts: a meta-analysis measuring cyber and traditional bullying. J. Adolesc. Health 55, 602–611. 10.1016/j.jadohealth.2014.06.007 25168105

[B34] Molchanov S. V. (2014). Empathy as the factor of moral dilemma solving in adolescence. Procedia Soc. Behav. Sci. 146, 89–93. 10.1016/j.sbspro.2014.08.091

[B35] Obermann M. L. (2013). Temporal aspects of moral disengagement in school bullying: crystallization or escalation? J. Sch. Violence 12, 193–210. 10.1080/15388220.2013.766133

[B36] Ortega-Ruiz R. (2010). Agresividad injustificada, bullying y violencia escolar. Madrid: Alianza Editorial.

[B37] Ortega-Ruiz R. (2020). Educación para el Desarrollo Sostenible: del proyecto cosmopolita a la ciberconvivencia. Investigación En La Escuela 100, 11–22. 10.12795/IE.2020.i100.02

[B38] Ortega-Ruiz R. Del Rey R. Casas J. A. (2016). Assessing bullying and cyberbullying: Spanish validation of EBIPQ and ECIPQ. Psicologia Educativa 22, 71–79. 10.1016/j.pse.2016.01.004

[B39] Perren S. Gutzwiller-Helfenfinger E. Malti T. Hymel S. (2012). Moral reasoning and emotion attributions of adolescent bullies, victims, and bully-victims. Br. J. Dev. Psychol. 30, 511–530. 10.1111/j.2044-835X.2011.02059.x 23039330

[B40] Pornari C. D. Wood J. (2010). Peer and cyber aggression in secondary school students: the role of moral disengagement, hostile attribution bias, and outcome expectancies. Aggress. Behav. 36, 81–94. 10.1002/ab.20336 20035548

[B41] Pozzoli T. Gini G. Vieno A. (2012). Individual and class moral disengagement in bullying among elementary school children. Aggress. Behav. 38, 378–388. 10.1002/ab.21442 22778018

[B42] Preckel K. Kanske P. Singer T. (2018). On the interaction of social affect and cognition: empathy, compassion and theory of mind. Curr. Opin. Behav. Sci. 19, 1–6. 10.1016/j.cobeha.2017.07.01033421860

[B43] Rodríguez-Hidalgo A. J. Calmaestra J. Casas J. A. Ortega-Ruiz R. (2019). Ethnic-cultural bullying versus personal bullying: specificity and measurement of discriminatory aggression and victimization among adolescents. Front. Psychol. 10:46. 10.3389/fpsyg.2019.00046 30774605 PMC6367499

[B44] Romera E.va M. Ortega-Ruiz R. Runions K. Falla D. (2020). Moral disengagement strategies in online and offline bullying. Psychosoc. Interv. 30, 85–93. 10.5093/pi2020a21

[B45] Romera E. M. Casas J. A. Gómez-Ortiz O. Ortega-Ruiz R. (2019a). Moral domain as a risk and protective factor against bullying. An integrating perspective review on the complexity of morality. Aggress. Violent Behav. 45, 75–82. 10.1016/j.avb.2018.07.005

[B46] Romera E. M. Ortega-Ruiz R. Rodríguez-Barbero S. Falla D. (2019b). How do you think the victims of bullying feel? A study of moral emotions in primary school. Front. Psychol. 10, 1–11. 10.3389/fpsyg.2019.01753 31428018 PMC6690008

[B47] Runions K. C. Bak M. (2015). Online moral disengagement, cyberbullying, and cyber-aggression. Cyberpsychol. Behav. Soc. Netw. 18, 400–405. 10.1089/cyber.2014.0670 26167839

[B48] Runions K. C. Shaw T. Bussey K. Thornberg R. Salmivalli C. Cross D. S. (2019). Moral disengagement of pure bullies and bully/victims: shared and distinct mechanisms. J. Youth Adolesc. 48, 1835–1848. 10.1007/s10964-019-01067-2 31278567

[B49] Silke C. Brady B. Boylan C. Dolan P. (2018). Factors influencing the development of empathy and pro-social behaviour among adolescents: a systematic review. Child. Youth Serv. Rev. 94, 421–436. 10.1016/j.childyouth.2018.07.027

[B50] Singleton R. Straits B. (2004). Approaches to Social Research. Oxford: Oxford University Press.

[B51] Smith P. K. López-Castro L. Robinson S. Görzig A. (2019). Consistency of gender differences in bullying in cross-cultural surveys. Aggress. Violent Behav. 45, 33–40. 10.1016/j.avb.2018.04.006

[B52] Stavrinides P. Georgiou S. Theofanous V. (2010). Bullying and empathy: a short-term longitudinal investigation. Educ. Psychol. 30, 793–802. 10.1080/01443410.2010.506004

[B53] Tampke E. C. Fite P. J. Cooley J. L. (2020). Bidirectional associations between affective empathy and proactive and reactive aggression. Aggress. Behav. 46, 317–326. 10.1002/ab.21891 32227484 PMC8075037

[B54] Thornberg R. Jungert T. (2014). School bullying and the mechanisms of moral disengagement. Aggress. Behav. 40, 99–108. 10.1002/ab.21509 24496999

[B55] Thornberg R. Wänström L. Pozzoli T. Hong J. S. (2019). Moral disengagement and school bullying perpetration in middle childhood: a short-term longitudinal study in Sweden. J. Sch. Violence 18, 585–596. 10.1080/15388220.2019.1636383

[B56] Vachon D. D. Lynam D. R. (2013). The (Non)relation between empathy and aggression: surprising results from a meta-analysis five factor machiavellianism inventory view project hunting for the true TriPM view project. Psychol. Bull. 140, 751–773. 10.1037/a0035236 24364745

[B57] van Noorden T. H. J. Haselager G. J. T. Cillessen A. H. N. Bukowski W. M. (2015). Empathy and involvement in bullying in children and adolescents: a systematic review. J. Youth Adolesc. 44, 637–657. 10.1007/s10964-014-0135-6 24894581

[B58] Walters G. D. Espelage D. L. (2018). Resurrecting the empathy–bullying relationship with a pro-bullying attitudes mediator: the lazarus effect in mediation research. J. Abnorm. Child Psychol. 46, 1229–1239. 10.1007/s10802-017-0355-9 29038937

[B59] Weisz E. Cikara M. (2020). Strategic regulation of empathy. Trends Cogn. Sci. 25, 213–227. 10.1016/j.tics.2020.12.002 33386247

[B60] Wen Z. Fan X. (2015). Monotonicity of effect sizes: questioning kappa-squared as mediation effect size measure. Psychol. Methods 20, 193–203. 10.1037/met0000029 25664380

[B61] Widom C. S. (1989). The cycle of violence. Science 244, 160–166. 10.1126/science.2704995 2704995

[B62] Williford A. Boulton A. J. Forrest-Bank S. S. Bender K. A. Dieterich W. A. Jenson J. M. (2016). The effect of bullying and victimization on cognitive empathy development during the transition to middle school. Child Youth Care Forum 45, 525–541. 10.1007/s10566-015-9343-9

[B63] Zych I. Llorent V. J. (2019). Affective empathy and moral disengagement related to late adolescent bullying perpetration. Ethics Behav. 29, 547–556. 10.1080/10508422.2018.1521282

[B64] Zych I. Ttofi M. M. Farrington D. P. (2019). Empathy and callous–unemotional traits in different bullying roles: a systematic review and meta-analysis. Trauma Violence Abuse 20, 3–21. 10.1177/1524838016683456 30803395

