# Supplementary material for: Aggression, Moral Disengagement and Empathy. A Longitudinal Study Within the Interpersonal Dynamics of Bullying
Source: Front Psychol. 2021 Sep 10;12:703468. doi: 10.3389/fpsyg.2021.703468 (PMC8461074; doi:10.3389/fpsyg.2021.703468)
Supplement: Supplementary file 1 [file Table_1.pdf]

SUPPLEMENTAL MATERIAL (TABLES AND FIGURES)

SUPPLEMENTAL TABLES

Supplemental Table 1.

	1	2	3	4	5	6	7	8	9	10	11	12	13	14	15	16	17	18	19	20	21	22
1. BP T1																						
2. CR T1	.49**																					
3. MR T1	.29**	.55**																				
4. DC T1	.38**	.62**	.49**																			
5. DH T1	.39**	.71**	.57**	.57**																		
6. CE T1	-.17**	-.20**	-.12**	-.22**	-.17**																	
7. AE T1	-.19**	-.30**	-.16**	-.26**	-.27**	.43**																
8. BP T2	.43**	.29**	.21**	.25**	.25**	-.12**	-.18**															
9. CR T2	.35**	.62**	.34**	.38**	.45**	-.18**	-.30**	.43**														
10. MR T2	.16**	.30**	.46**	.24**	.29**	-.06*	-.12**	.29**	.54**													
11. DC T2	.25**	.39**	.29**	.42**	.31**	-.14**	-.21**	.36**	.64**	.52**												
12. DH T2	.29**	.47**	.36**	.33**	.54**	-.15**	-.24**	.38**	.70**	.58**	.62**											
13. CE T1	-.17**	-.22**	-.12**	-.19**	-.19**	.54**	.32**	-.19**	-.24**	-.09**	-.24**	-.25**										
14. AE T1	-.20**	-.32**	-.18**	-.22**	-.25**	.32**	.69**	-.18**	-.31**	-.11**	-.23**	-.25**	.41**									
15. BP T3	.31**	.26**	.17**	.18**	.20**	-.10**	-.11**	.42**	.32**	.21**	.26**	.26**	-.12**	-.12**								
16. CR T3	.34**	.60**	.33**	.35**	.46**	-.17**	-.23**	.29**	.66**	.37**	.40**	.50**	-.17**	-.27**	.40**							
17. MR T3	.19**	.35**	.47**	.27**	.34**	-.09**	-.09**	.25**	.39**	.56**	.34**	.42**	-.12**	-.12**	.30**	.58**						
18. DC T3	.20**	.37**	.26**	.35**	.32**	-.13**	-.21**	.20**	.43**	.31**	.45**	.42**	-.16**	-.21**	.28**	.65**	.56**	.				
19. DH T3	.26**	.45**	.31**	.31**	.52**	-.12**	-.21**	.27**	.51**	.38**	.38**	.62**	-.18**	-.23**	.37**	.74**	.62**	.66**				
20. CE T1	-.19**	-.21**	-.10**	-.17**	-.16**	.50**	.31**	-.16**	-.20**	-.05*	-.16**	-.19**	.56**	.35**	-.15**	-.17**	-.09**	-.18**	-.18**			
21. AE T1	-.20**	-.30**	-.16**	-.22**	-.22**	.30**	.64**	-.14**	-.29**	-.14**	-.22**	-.25**	.31**	.70**	-.14**	-.29**	-.15**	-.26**	-.24**	.46**		
22. Age	.08**	.15**	.06*	.11**	.10**	-.10**	-.12**	.04	.15**	.04	.09**	.09**	-.10**	-.09**	.03	.09**	-.003	.07**	.06*	-.09**	-.09**	
23. Gender	-.13**	-.26**	-.10**	-.19**	-.20**	.23**	.33*	-.15**	-.27**	-.07**	-.16**	-.18**	.24**	.36**	-.12**	-.25**	-.07**	-.19**	-.20**	.23**	.36**	.01

Note: BP, Bullying perpetration; CR, Cognitive restructuring; MR, Minimizing responsibility; DC, Distorting consequences; DH, Dehumanizing; CE, Cognitive Empathy, AE, Affective Empathy; * $p < .05$; ** $p < .001$. Gender was dummy coded such that 1 = boy, 2 = girl
